# Patients' perception towards large language models in otorhinolaryngology, head and neck surgery: a single-centre survey

**DOI:** 10.3389/fdgth.2026.1849286

**Published:** 2026-07-08

**Authors:** Christoph R. Buhr, Andrew Blaikie, Harry Smith, Tom Kelsey, Christian Ruckes, Christoph Matthias, Sebastian Kuhn, Jonas Eckrich

**Affiliations:** 1 Department of Otorhinolaryngology, University Medical Center of the Johannes Gutenberg-University Mainz, Mainz, Rhineland-Palatinate, Germany; 2 School of Medicine, University of St Andrews, St Andrews, United Kingdom; 3 School of Computer Science, University of St Andrews, St Andrews, United Kingdom; 4 Interdisciplinary Center for Clinical Trials (IZKS), University Medical Center of the Johannes Gutenberg-University Mainz, Mainz, Rhineland-Palatinate, Germany; 5 Institute for Digital Medicine Philipps-University Marburg and University Hospital of Giessen and Marburg, Marburg, Germany

**Keywords:** artificial intelligence (AI), head and neck, large language models (LLMs), ORL, otorhinolaryngology, patient perception

## Abstract

**Objectives:**

Large language models (LLMs) are increasingly discussed for use in clinical practice. Beyond their performance, patients' acceptance is crucial for their implementation. We investigated ORL-HNS patients' familiarity with AI/LLMs, use patterns, and trust in LLM-based medical information and recommendations.

**Methods:**

In this single-centre prospective survey at a German university hospital, ORL-HNS patients with and without malignant disease completed a 15-item questionnaire.

**Results:**

A total of 123 patients, 20 (16%) with and 103 (84%) without malignant disease, participated in the study. Most patients were familiar with the term AI (96%, *n* = 118) and LLMs (78%, *n* = 96). Overall, 72/123 (59%) reported using LLMs. One third (33%, *n* = 40) retrieved “Health information”, rating the LLMs with median Likert scores for comprehensibility 5 [IQR 4, 6], conciseness 5 [IQR 3, 6] and coherence 5 [IQR 3, 6]. However, perceived medical accuracy received a median rating of 4 [IQR 3, 5], significantly lower than comprehensibility (*p* < 0.05). With respect to the confidence in the recommendations exclusively by LLMs [median 2 (IQR 2, 3.5)] received significantly lower ratings than doctors [median 5 (IQR 5, 6)] and doctors also using LLMs [median 5 (IQR 4, 6)], *p* < 0.0001 respectively.

**Conclusion:**

ORL-HNS patients are largely familiar with LLMs and frequently use them, but their trust and confidence regarding health information provided by LLMs alone is limited. Patients show the greatest confidence in doctors' recommendations. Yet they reported similar confidence in physician recommendations and physician recommendations supported by LLMs, suggesting that clinician-led LLM use may be acceptable to many patients.

## Background

The use of artificial intelligence (AI) and, in particular, large language models (LLMs) in medicine is becoming increasingly common. Various studies have shown a wide range of possible applications in the field of Otorhinolaryngology, Head and Neck Surgery (ORL-HNS) (1). Areas of application in ORL-HNS include patient information (2), diagnosis (3) and decision-making (4, 5). Beyond the frequently evaluated performance of LLMs, patients' acceptance is essential for the implementation of LLMs in clinical practice. To date, little is known about ORL-HNS patients' familiarity with LLMs, how they use them, and how much they trust LLM-based medical information.

### Familiarity and use of AI and LLMs

In a recent study, up to 96% of adults (18+ years) in the UK self-reported awareness of the term AI (6). These results are consistent with data from the US from 2024. Here, 90% of the interviewed Americans (18–65+ years) had heard of the term AI, while in contrast, only 12% were familiar with the term LLM (7). Both studies are focused on people's knowledge and attitude towards AI in general. LLMs, in particular, are becoming part of everyday life. Chatterji et al. analysed a random selection of messages sent to ChatGPT, concluding that users apply for private (73%) and work-related (27%) queries, with both areas increasing (8). According to this study, the three most common conversations are dealing with practical guidance (29%), seeking information (24%), and writing (24%). Writing showed to be popular in professional occupations like education (50%) and health care (49%). Another recent study underscores the relevance of LLM use in academic research and publishing of clinical research (9). Beyond research, AI is commonly used for personal health monitoring purposes in mobile phones and sports watch apps. One report showed that around 73% of smartphone owners regularly used used at least one health app (10). These findings underline the awareness of AI in the general and healthy population, and the relevance of LLMs in personal health and pursuing medical information.

### LLMs for medical information and patients' confidence

In a recent report conducted by the Bitkom association in Germany, 45% of Germans said they would ask AI chatbots about symptoms and health issues (10). A similar study among patients with disease in the field of rheumatology from 2025 revealed that 19% of the participating patients use AI to find information on their disease (11). Despite LLMs' ability to answer medical queries (2), studies highlight patients' ongoing scepticism towards LLMs' competence on health-related issues (12, 13). Within a study conducted by Reis et al., the researchers mislabelled the source of the medical advice (“AI”, “human physician”, “human + AI”) (12). Although patients received the same information, “AI”- and “human + AI”-labelled advice was evaluated as significantly less reliable and less empathetic compared to “human”-labelled advice. Shekar et al. (14), analysed medical responses that were written by a medical doctor on an online healthcare platform or generated by some LLMs'. They reported that participants could not effectively distinguish between doctors and LLMs' responses (14). The authors asked the participants to evaluate the LLMs' responses in terms of medical content (high or low accuracy). Interestingly, the participants rated low-accuracy LLM responses as being similarly good in terms of validity, confidence, and completeness/satisfactory as the contributions made by the doctors. Moreover, participants stated a high tendency to follow the potentially harmful medical advice provided by the LLMs. These findings underline that both trust and risk perception may be important barriers to safe LLM use for health information.

For patients in ORL-HNS, there is currently very little data available on patient usage behaviour, confidence in the technology, and acceptance of LLM-augmented medical decision-making. Consequently, this prospective, single-centre study aims to examine the LLM usage behaviour of ORL-HNS patients and their attitudes towards its use in medical decision-making in more detail. In ORL-HNS, malignant and non-malignant conditions can differ materially in complexity, decisional burden, perceived risk, and need for longitudinal information-seeking. Therefore, exploring whether trust and LLM use vary by malignant disease status has clinical relevance.

## Methods

Ethical approval was obtained from the ethics committee of the state medical association (request/approval number: 2024-17946).

### Study design

Patients with and without malignant disease from ORL-HNS undergoing treatment within our department were invited to participate in this study. Adult patients from ORL-HNS capable of the German language were recruited from 13.08.2025 to 1.11.2025. Minors were not included. After providing written informed consent, patients completed an anonymous paper- or web-based questionnaire (LimeSurvey, Hamburg, Germany). Participants did not receive any remuneration.

**Figure 1:**
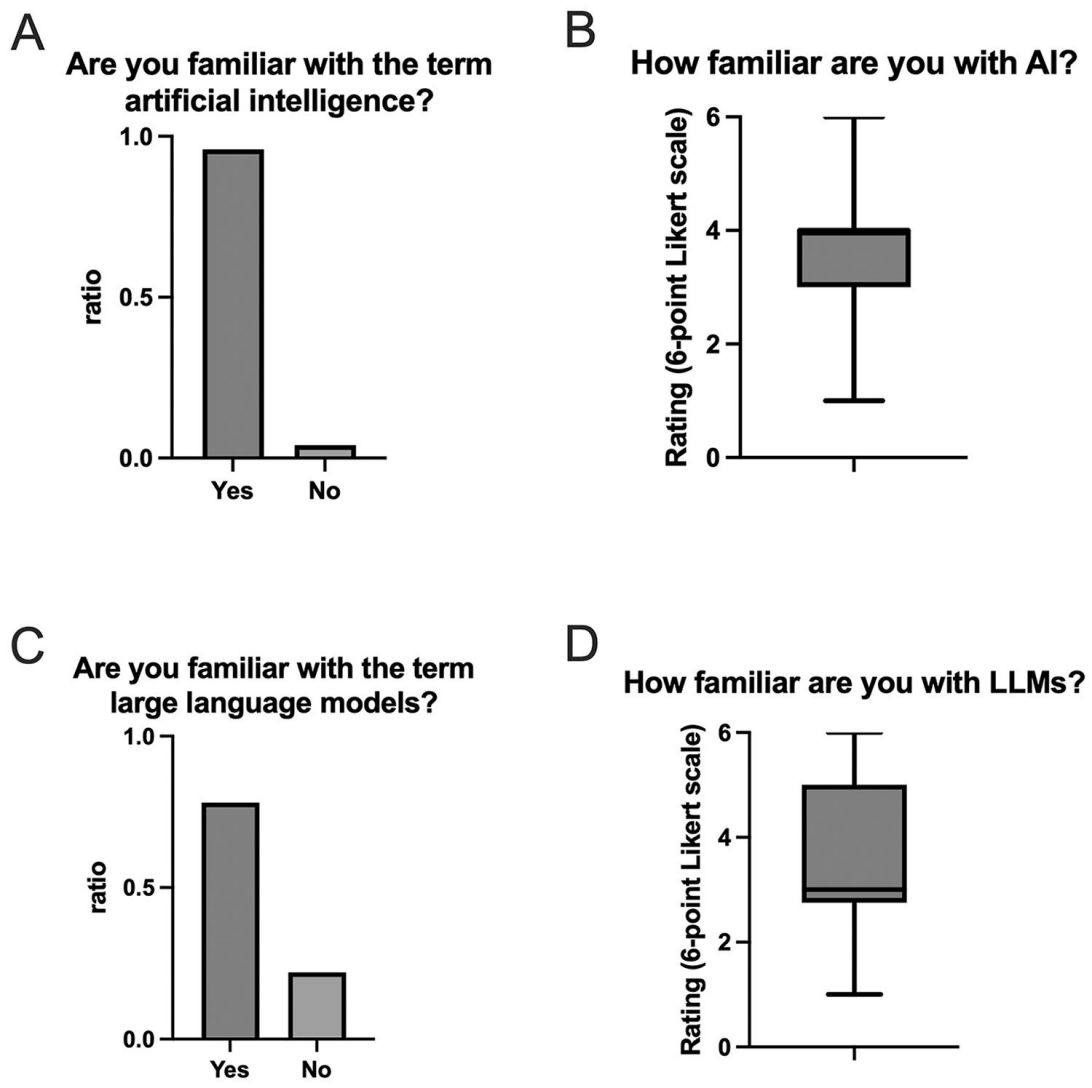
Familiarity with artificial intelligence (AI) & large language models (LLMs), results visualised for all participants, **(A,C)** shown as ratio, **(B,D)** shown as box plots, *y*-axis resembling the Likert rating (1 = very low to 6 very high).

**Figure 2:**
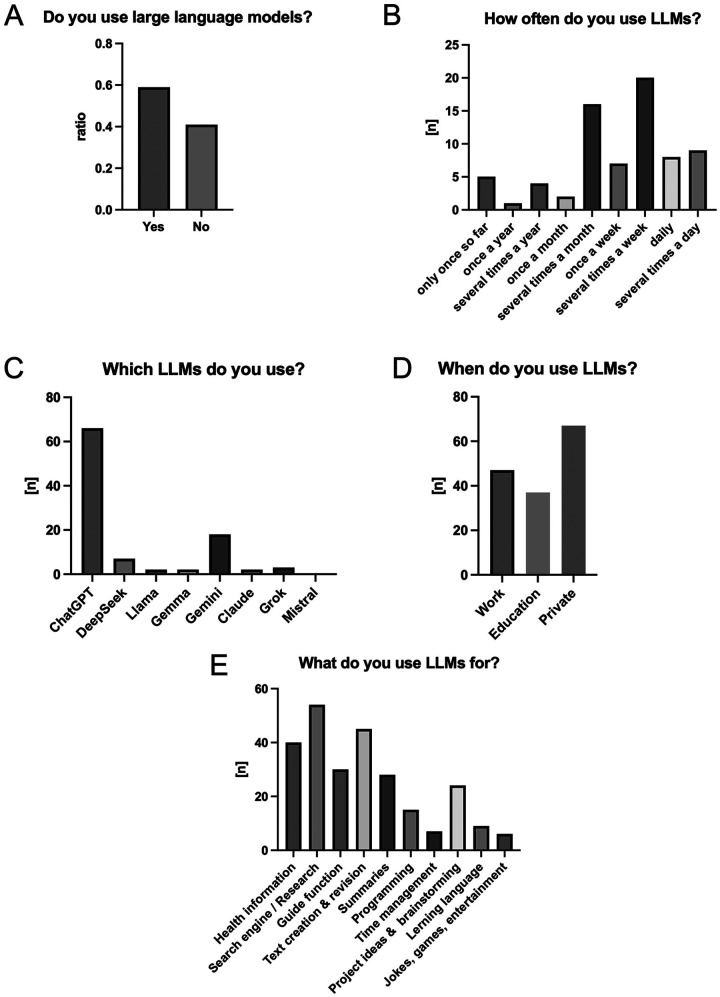
General large language models (LLMs) application patterns, results visualised for all participants, **(A)** shown as ratio, other data visualised as absolute values [n]. **(C–E)** Multiple responses were allowed where applicable.

**Figure 3:**
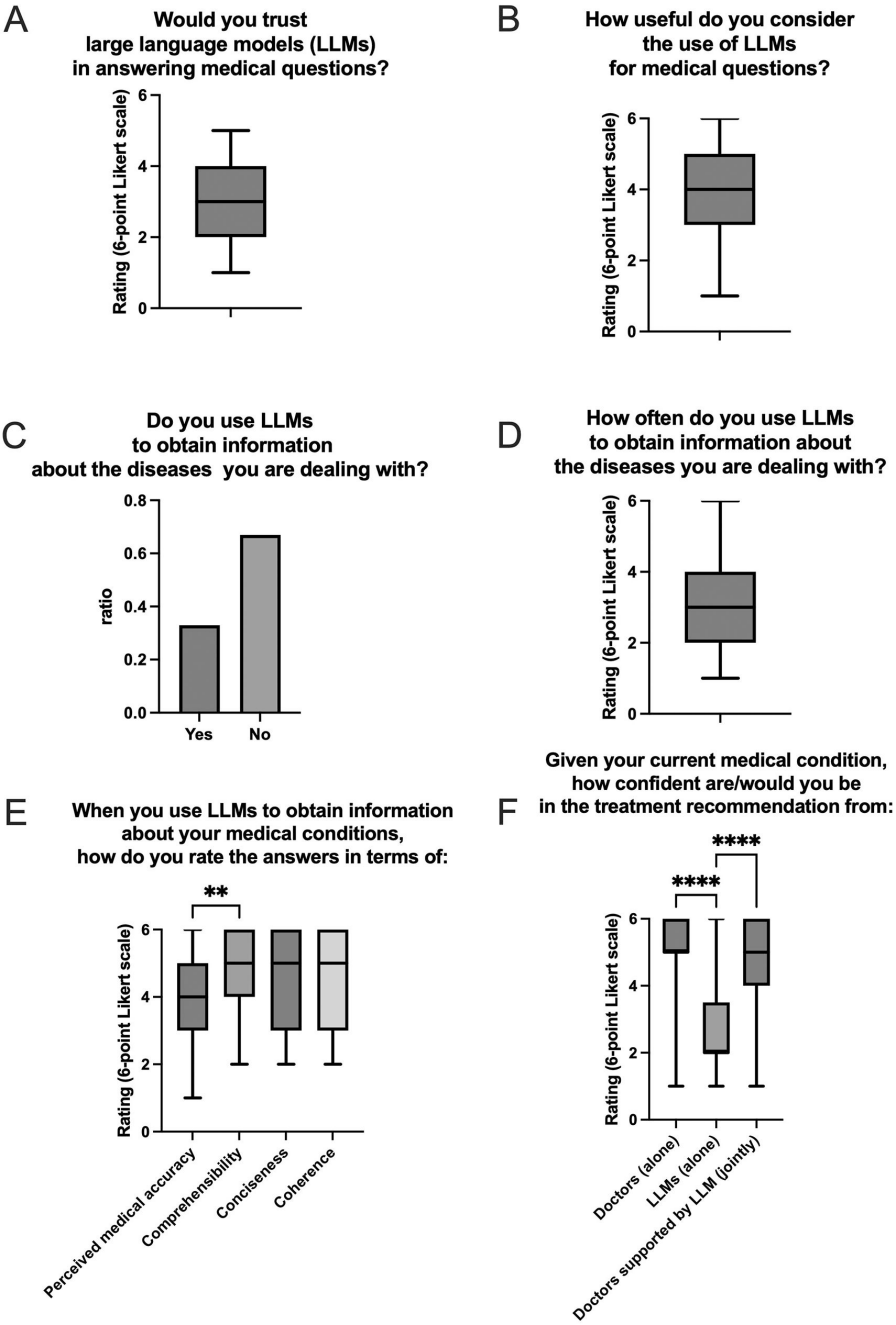
Large language model (LLM) application patterns for medical purposes, data shown as box plots with exception of **(C)** providing absolute values [n]. Pairwise comparison in **(E)** and **(F)** by Dunn's multiple comparison test. Pairwise comparison with *p* > 0.05 is not displayed for the sake of clarity. ** ≙ *p* < 0.005, *** ≙ *p* < 0.0005.

The questionnaire comprised 15 items, 4 on familiarity with AI/LLMs (Figure 1), 5 on general LLM application patterns (Figure 2) and 6 on LLM use for medical purposes (Figure 3). Question types include binary questions (yes/no), 6-point Likert rating (1 = very poor and 6 = excellent), and multiple choice depending on the subsequent question (see Figures 1–3).

### Statistical analysis

Statistical analyses were performed using GraphPad Prism Version 10.6.1. for macOS (GraphPad Software, La Jolla, CA, USA). The data did not meet normality assumptions, as confirmed by the D'Agostino and Pearson test. ns ≙ > 0.05, * ≙ *p* < 0.05, ** ≙ *p* < 0.005, *** ≙ *p* < 0.0005. Inferential statistics for binary and multiple-choice questions were performed by Fisher's exact test, Likert scaled questions were analysed for multiple group comparison using the Kruskal–Wallis test and *post-hoc* pairwise analysis using Dunn's multiple comparison test. Subgroup comparison between tumour and non-tumour patients was performed by the Mann–Whitney test. Spearman correlation coefficients were calculated for Likert scaled questions (See Supplemenaty Figure S1).

## Results

From 13.08.2025 to 1.11.2025, 123 patients participated in the study, 19 patients refused to participate, which corresponds to a participation rate of 87%. As an approximate parameter of severity of disease, a subgroup analysis of malignant diseases was conducted. Among patients, 103 (84%) did not have malignant disease and 20 (16%) had malignant disease from ORL-HNS. A total of 32% (*n* = 39) participants were female and 68% (*n* = 84) were male. 14% (*n* = 17) of the participants were under 30 years of age, 49% (*n* = 60) were between 30 and 60, and 37% (*n* = 46) were over 60 years of age.

### Familiarity with artificial intelligence (AI) & large language models (LLMs)

A majority of patients [96% (*n* = 118)] stated to be familiar with the term AI (Figure 1A), rating their familiarity with a median of 4 (IQR 3, 4) on a 6-point Likert scale (Figure 1B). A slightly smaller portion of 78% (*n* = 96) of patients confirmed to be familiar with the term „LLM“ (Figure 1C), rating their familiarity with a median of 3 (IQR 2.75, 5) (Figure 1D).

### General LLM application patterns

Fifty-nine percent (*n* = 72) of participants reported using LLMs (Figure 2A). A majority of these participants stated to use LLMs “several times a week” 16% (*n* = 20) or “several times a month” 13% (*n* = 16). Some LLM-using participants even stated to use LLMs “several times a day” 7% (*n* = 9) and “daily” 7% (*n* = 8) (Figure 2B). Among participants, ChatGPT is the most frequently used LLM 54% (*n* = 66), followed by Gemini 15% (*n* = 18) (Figure 2C). Most participants use LLMs for private purposes 54% (*n* = 67), followed by work 39% (*n* = 47) and education purposes 30% (*n* = 37) (Figure 2D). “Search engine/Research” 44% (*n* = 54) was most frequently selected for LLMs application, followed by “Text creation & revision” 37% (*n* = 45) and “Health information” 33% (*n* = 40) (Figure 2E).

### LLM application patterns for medical purposes

Regarding LLMs' application for medical purposes, participants rated their confidence with a median of 3 [IQR 2, 4] (Figure 3A) and their usefulness for medical queries with a median of 4 [IQR 3, 5] (Figure 3B). Overall, 32% (*n* = 40) stated LLMs use to obtain information for the disease they are dealing with (reason of treatment within our clinic) (Figure 3C). With respect to the frequency of using LLMs for this specific purpose, the participants indicate a median of 3 [IQR 2, 4] (Figure 3D). When using the LLMs regarding their medical condition, participants rated perceived medical accuracy with a median of 4 [IQR 3, 5], comprehensibility 5 [IQR 4, 6], conciseness 5 [IQR 3, 6] and coherence 5 [IQR 3, 6]. Inferential statistics showed a significant difference between the rating for perceived medical accuracy and comprehensibility *p* < 0.005 in the *post-hoc* Dunn's multiple comparison test. All other pairwise comparisons did not reach significance (Figure 3E).

With respect to the confidence in the recommendation, doctors achieved a median of 5 (IQR 5, 6), LLMs 2 (IQR 2, 3.5) and doctors supported by LLM (i.e., doctors' recommendations made with LLM support) received a median rating of 5 (IQR 4, 6). Inferential statistics showed significant differences between doctors and LLMs as well as between LLMs and doctors supported by LLM (jointly) *p* < 0.0001, respectively (Figure 3F).

## Discussion

Various studies have investigated the performance of AI and LLMs in ORL-HNS (1). Patients' acceptance is, however, also crucial for implementation in everyday clinical practice. There is a growing number of studies on assessing this area, all of which are outside the field of ORL-HNS (6–8, 10–15). We therefore surveyed ORL-HNS patients' familiarity with AI/LLMs, their use patterns, and their confidence in LLM-based medical information and recommendations.

### Familiarity and use of AI and LLMs

In this cohort, most patients were familiar with the terms AI (96%) and LLM (78%) (Figures 1A,C). The numbers determined for AI familiarity are similar to those published in previous surveys from the UK and the US, with 96% of British (6) and 90% of American (7) respondents stating to be familiar with the term AI. Familiarity with the common AI term LLM was much lower, reaching 12% among Americans in literature (7). The difference between AI and LLMs may be related to the fact that the term LLMs is used less frequently in the media than AI. A Google search using the terms “AI” and “LLM” during the drafting of the manuscript (December 2025) yielded 8.7 billion results for AI but only 64.5 million results for LLM. Unsurprisingly, therefore, in our study, familiarity was reported to be higher for AI [median 4, (IQR 3, 4)] than for LLMs [median 3, (IQR 2.75, 5)] (Figures 1B,C). Despite this, the largest proportion of respondents used LLMs “several times a week” (16%), “at least once a day” (14%) or “several times a month” (13%) (Figure 2B), primarily for “private” purposes (55%) (Figure 2D). A survey from the USA shows higher usage figures than in our study (32% at least once a day), but very similar values for the usage context (52% private use) (16). ChatGPT is by far the most frequently used (55%), followed by Gemini (15%) (Figure 2C). This chronology corresponds with a survey among U.S. Americans finding that 72% have used ChatGPT, while 50% have used Google's Gemini and 39% have used Microsoft's Copilot (16). In terms of specific use, “health information” ranked third 33%, directly after “search engine/research” (44%) and “text creation & revision” (37%) (Figure 2E). These estimates are similar to previously published studies and highlight the high relevance of LLMs for patients obtaining health information (8, 10).

### LLMs for medical information and patients' confidence

To develop a better understanding of how patients use LLMs for health information, we asked patients about their confidence in LLMs and their perceived usefulness when it comes to medical questions. Our patients gave a median rating of 3 [IQR 2, 4] for confidence and a median rating of 4 [IQR 3, 5] for usefulness (Figures 3A,B). Approximately one-third of patients (32%) use LLMs to obtain information about their current illness, with a median frequency of 3 [IQR 2, 4] (Figure 3D). When asked to evaluate the LLMs' answers to health questions in more detail, the categories comprehensibility 5 [IQR 4, 6], conciseness 5 [IQR 3, 6] and coherence 5 [IQR 3, 6] received similar responses. Perceived medical accuracy, on the other hand, was rated notably lower 4 [IQR 3, 5]. The difference between comprehensibility and perceived medical accuracy was statistically significant (*p* < 0.05) (Figure 3E). Why comprehensibility, conciseness and coherence of the LLMs' answers were rated similarly remains unclear. It might be possible that patients do not perceive the distinction as relevant and subsume the different items under comprehensibility. However, the poor performance in terms of perceived medical accuracy is interesting, especially when considering the following question. Here, respondents were asked how much confidence they have in the recommendations of doctors, LLMs and doctors supported by LLM (jointly). LLMs [median 2, (IQR 2, 3.5)] received significantly worse ratings than doctors [median 5, (IQR 5, 6)] and doctors supported by LLM (jointly) [median 5, (IQR 4, 6)] (Figure 3F). However, our survey cannot clarify whether patients have little confidence in LLMs due to bad experiences (e.g., misinformation) or whether causality is reversed, with the lack of confidence causing the poor rating for perceived medical accuracy. Nonetheless, the literature indicates that patients' perception of whether advice originates from AI or humans does influence their perceived quality (12, 13). Whether this can be attributed to the fact that LLMs are perceived as a relatively new and untried technology or negative media coverage related to large AI companies being involved in politics has yet to be determined.

### Implications of the study

Although the patients we surveyed were mostly familiar with AI and LLMs, using them regularly, there is still a certain degree of scepticism towards AI for medical applications, especially when directly compared to doctors (Figure 3F). This is consistent with findings from other studies and has implications and opportunities for the implementation of AI in everyday clinical practice (12, 13).

While in one study the patients' ratings for AI and doctor responses received similar ratings (14), in another study the AI's results were rated worse than the doctors' responses (12). The difference can be explained in how the answers were labelled. While in one study the author was blinded (the AI achieved similar results to the doctors) (14), in the other study the same answers were labelled differently (“AI”, “human physician”, “human + AI”) and the doctors received better ratings (12). Accordingly, it seems to make a difference for patients' perception whether patients assume that the answer comes from a doctor or an AI. This is a highly relevant finding for the implementation of AI in medicine.

Our study shows that patients have enormous confidence in doctors (Figure 3F). Doctors and doctors supported by LLM (jointly) received significantly better ratings each in terms of confidence than LLMs. Even between doctors and doctors supported by LLM (jointly), doctors proved to be superior, albeit not significantly. One could conclude that some patients apparently want treatment exclusively from doctors or are sceptical about LLMs, which may have a negative influence on medical decision-making. In any case, the patient group studied prefers a joint decision by doctor and LLM to a decision made solely by LLMs. Doctors supported by AI such as LLMs have rather more than less access to information, so the lower rating by patients can only be explained due to concerns about a potential “Automation Bias” (17). In other words, the fear that doctors will rely solely on AI and become inattentive. The results of the study underscore the high level of confidence placed in doctors, which should be strengthened by responsible behaviour on the part of doctors and not undermined by negligent actions. In this way, doctors can strengthen their profession, because patients' perspectives can also change. AI can take on tedious tasks, liberate time, increase precision, and allow more time for interpersonal interaction (18–20). By following this approach and ensuring that patients and doctors receive adequate training, we can further advance medicine using AI.

Despite the monocentric design and the limited sampling size, results regarding awareness of AI and LLMs and the general use of LLMs are consistent with the results of previous studies, which underscores the significance of the present findings (6–8, 10). The present study did not show significant differences between tumour and non-tumour patients' perception of LLMs and doctors' medical advice (Figures 3E,F). As the sub group of tumor patients was rather small (*n* = 20), future studies should investigate this aspect further. It is conceivable that the severity of the disease also influences confidence in AI with regard to information seeking and decision-making. Initial studies differentiating between acute and chronic disease groups in the analysis of patient confidence in AI exist (21). Here the authors find that trust in doctors was significantly higher for patients with acute conditions (*P* < .001), although the disease category differs for the studies, our malignant disease subgroup was small, and hence our results are hypothesis-generating rather than confirmatory. There are already a number of studies that focus specifically on the attitudes of cancer patients without benchmarking their perception against non-cancer patients (22–24). Nonetheless, the patient perspective in general has received little attention in the literature, but the implementation of AI in medicine can only take place if patients are taken into account, and this should be considered in the future.

### Limitations

This study has limitations. It was a single-centre survey with a relatively small subgroup of patients with malignant disease (*n* = 20), which limits subgroup comparisons. The subgroup analyses were pre-planned with respect to clinical motivation, but underpowered for strong inference. The questionnaire was study-specific, and its psychometric properties were not formally validated and its data consists of reported opinions rather than outcomes from clinical discussions. Given the questionnaire-based design and the option to complete the questionnaire online, self-report bias, digital literacy bias and influence by social desirability cannot be ruled out. As patients cannot sufficiently assess medical accuracy, patients' perceived accuracy was assessed. Health-related LLM use may include very different behaviours ranging from checking symptoms to asking about prognosis or seeking a second opinion. To better characterise the risk associated with patients' LLM consultations, future multicentre studies with validated instruments should distinguish between low-risk educational uses and high-risk medical decision-making. Another important field of future research is the examination of how disease severity, prior LLM experience, and disclosure of LLM use by clinicians influence patient trust and decision-making.

## Conclusion

In this ORL-HNS cohort, most patients were familiar with AI/LLMs and many used LLMs, including for health information. To our knowledge, no studies have yet evaluated the relevance of patient perception towards LLMs and AI in the field of ORL-HNS. Yet, perception towards AI in medicine in general may be well represented by this specialty since ORL-HNS is a field with a broad variety of diseases throughout all ages, sexes, ethnicities, and involves acute and chronic as well as mild and severe disease. Nevertheless, confidence in LLM-only recommendations was low. Physician recommendations were trusted most, and describing physicians as using LLMs as a supportive tool did not meaningfully reduce confidence. Implementing LLMs in clinical practice should therefore be clinician-led and accompanied by patient-facing communication, governance, and training to maintain trust.

## Data Availability

Due to the sensitive nature of the clinical data used in this study and in accordance with institutional and legal data protection requirements, the datasets generated and analyzed cannot be made publicly available. Requests to access the datasets should be directed to buhrchri@uni-mainz.de.
