# Supplementary material for: Patients' perception towards large language models in otorhinolaryngology, head and neck surgery: a single-centre survey
Source: Front Digit Health. 2026 Jul 8;8:1849286. doi: 10.3389/fdgth.2026.1849286 (PMC13388865; doi:10.3389/fdgth.2026.1849286)
Supplement: Supplementary file 1 [file Datasheet1.pdf]

Appendix

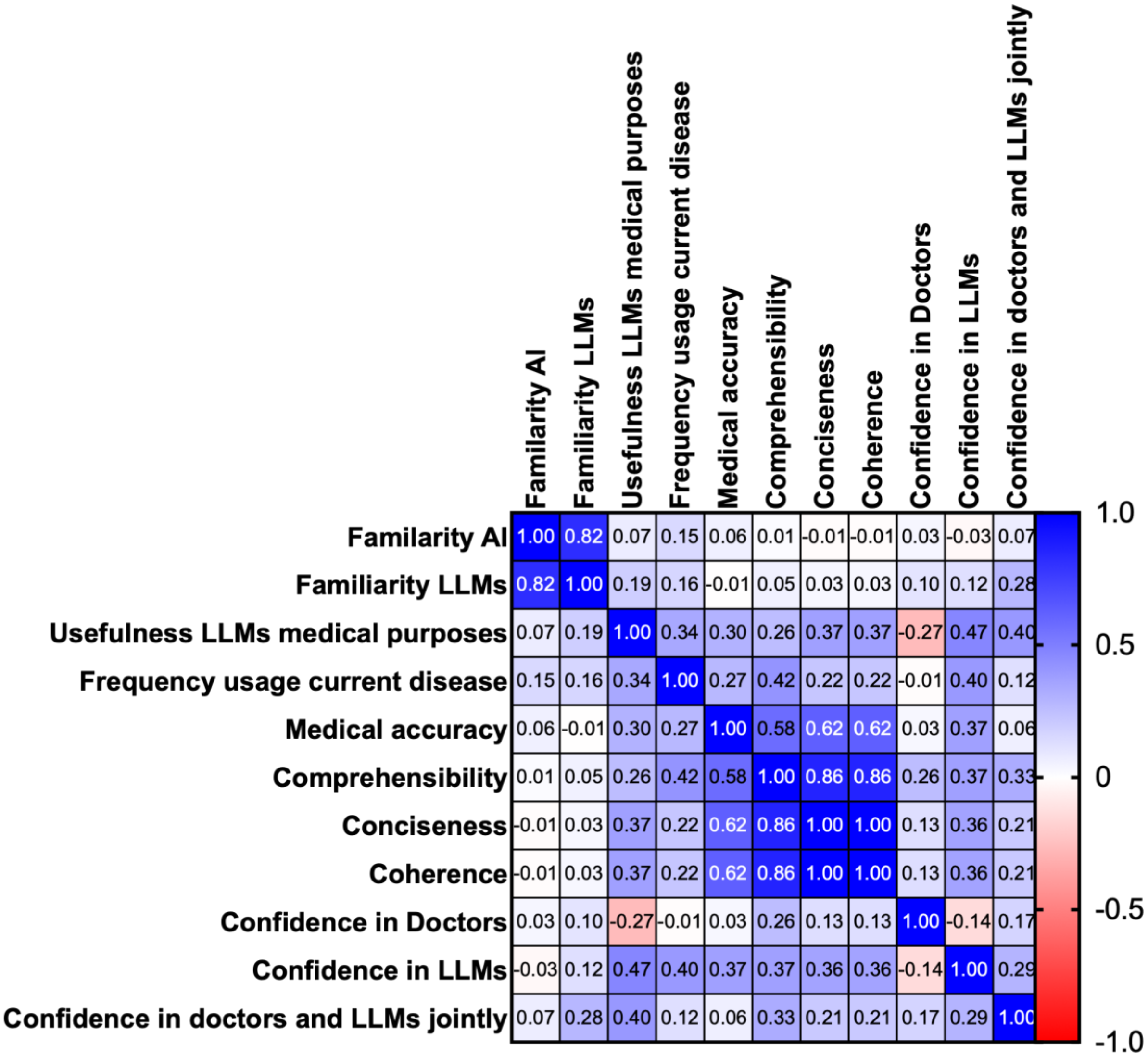

Appendix Figure 1: Spearman correlation coefficients for Likert scaled questions visualized as heatmap.

Questionnaire (Translated from German to English with deepl.com)

Part A: Knowledge of artificial intelligence (AI) and large language models (LLMs)

A1. Are you familiar with the term ‘artificial intelligence’?
(single choice)
yes/no

A2. How familiar are you with the topic of artificial intelligence?
(single choice)
Likert scale: 1 (not at all familiar) – 6 (very familiar)

A3. Have you ever heard of large language models such as ChatGPT?
(single choice)
yes/no

A4. How familiar are you with the topic of large language models, such as ChatGPT?
(single choice)
Likert scale: 1 (not at all familiar) – 6 (very familiar)

Part B: Use of Large Language Models (LLMs)

B1. Do you use large language models such as ChatGPT?
(single choice)
yes/no

B2. How often do you use large language models such as ChatGPT?
(single choice)
So far, only once
Once a year
Several times a year
Once a month
Several times a month
Once a week
Several times a week
Daily
Several times a day

B3. Which large language models do you use?
(multiple choice)
ChatGPT
DeepSeek
Llama
Gemma
Gemini
Claude
Grok
Mistral
Other (free text)

B4. When do you use large language models such as ChatGPT?
(multiple choice)
Work
Education
Personal

B5. What do you use large language models such as ChatGPT for?
(multiple choice)
Health information
Search engine / Research
Guide section
Copywriting & editing
Summaries
Programming help
Time management & planning
Project ideas & brainstorming
Language learning
Jokes, puzzles, games
Other (free text)

Part C: Trust in Large Language Models

C1. Would you trust large language models to answer medical questions?
(single choice)
Likert scale: 1 (not at all familiar) – 6 (very familiar)

C2. Do you think the use of large language models for medical questions is a good idea?
(single choice)
Likert scale: 1 (not at all familiar) – 6 (very familiar)

Part D: The use of large language models in relation to your current condition

D1. Do you use large language models to obtain information about the conditions you are dealing with?
(single choice)
yes/no

D2. How often do you use large language models to find out about the conditions you have?
(single choice)
Likert scale: 1 (very rare) – 6 (very often)

D3. If you use large language models to obtain information about your medical conditions, how do you assess the responses in terms of:

Medical accuracy (Do you consider the information to be factually correct?)
(single choice)
Likert scale: 1 (very poor) - 6 (very good)

Clarity (Is the information easy for you to understand?)
(single choice)
Likert scale: 1 (very poor) - 6 (very good)

Conciseness (Is the information concise and to the point?)
(single choice)
Likert scale: 1 (very poor) - 6 (very good)

Contextual coherence (Is the connection between the pieces of information clear to you?)
(single choice)
Likert scale: 1 (very poor) - 6 (very good)

D4. Given your current medical condition, how much confidence do you have/would you have in the treatment recommendation from:

Doctors only (on their own)
(single choice)
Likert scale: 1 (very low) - 6 (very high)

Large language models only (on their own)
(single choice)
Likert scale: 1 (very low) - 6 (very high)

Doctors supported by large language models (jointly)
(single choice)
Likert scale: 1 (very low) - 6 (very high),
Part E: Personal details

E1. Please state your gender
(single choice)
Female
Male
Other

E2. How old are you?
(single choice)
Under 30
30–60
Over 60
